# How does the medical graduates' self-assessment of their clinical competency differ from experts' assessment?

**DOI:** 10.1186/1472-6920-13-24

**Published:** 2013-02-13

**Authors:** Fatima Taleb Abadel, Abdulla Saeed Hattab

**Affiliations:** 1 Community Medicine and Public Health Department, Faculty of Medicine and Health Sciences, University of Aden, Aden, Yemen

## Abstract

**Background:**

The assessment of the performance of medical school graduates during their first postgraduate years provides an early indicator of the quality of the undergraduate curriculum and educational process. The objective of this study was to assess the clinical competency of medical graduates, as perceived by the graduates themselves and by the experts.

**Methods:**

This is a hospital based cross-sectional study. It covered 105 medical graduates and 63 experts selected by convenient sampling method. A self-administered questionnaire covering the different areas of clinical competency constructed on a five-point Likert scale was used for data collection. Data processing and analysis were performed using the Statistical Package for Social Science (SPSS) 16.0. The mean, frequency distribution, and percentage of the variables were calculated. A non-parametric Kruskal Wallis test was applied to verify whether the graduates' and experts' assessments were influenced by the graduates' variables such as age, gender, experience, type of hospital, specialty and location of work at a *(p ≤ 0.05)* level of significance.

**Results:**

The overall mean scores for experts' and graduates' assessments were 3.40 and 3.63, respectively *(p= 0.035).* Almost 87% of the graduates perceived their competency as good and very good in comparison with only 67.7% by experts. Female and male graduates who rated themselves as very good were 33.8% and 25% respectively. More than 19% of the graduates in the age group > 30 years perceived their clinical competency as inadequate in contrast with only 6.2% of the graduates in the youngest age group. Experts rated 40% of the female graduates as inadequate versus 20% of males, *(p= 0.04)*. More than 40% of the graduates in younger age group were rated by experts as inadequate, versus 9.7% of the higher age group >30 years *(p = 0.03)*.

**Conclusion:**

There was a wide discrepancy between the graduates' self-assessment and experts' assessment, particularly in the level of inadequate performance. Graduates in general, and those of younger age groups in particular, tend to overestimate their clinical skills and competency.

## Background

Medical educators have a major responsibility to evaluate the clinical competency of medical students and residents and to provide them with timely and useful feedback to ensure their continued progress and correction of shortcomings. Despite the explosion of technological advances, the clinical competency of medical interviewing, physical examination, and counseling remain vital to the successful care of patients [1,2]. The successful completion of a medical school education should provide students with a level of knowledge and skills necessary to carry out a junior doctor’s daily duties at a hospital. While the level of training is usually evaluated in medical exams, it stands to reason that the results of these exams do not represent the whole truth of how well-prepared a medical student feels at doing a doctor’s job. In fact, different researchers demonstrated that exam results do not correlate with a resident’s level of confidence or feeling of preparedness [3,4].

The Accreditation Council for Graduate Medical Education (ACGME), the American Board of Internal Medicine (ABIM) and the Institute for International Medical Education (IIME) strongly endorse the evaluation of students and residents in these clinical competencies through direct observation [5-8]. Evaluation is at the heart of professionalism for the medical educator. Medical educators have a moral and professional obligation to ensure that any graduate leaving their training program has attained a minimum level of clinical competency to care for patients safely, effectively, and compassionately. Medical educators should not wait for the results of a standardized clinical competency exam or other examinations to learn whether their graduates possess sufficient clinical competencies. This responsibility cannot be abdicated to standardized patients, licensing boards, or computer simulators. Graduates recognize the importance of these clinical competencies; they also recognize that they are not always adequately prepared to care for patients after graduating from a residency program [9,10].

Self-assessment is critical to the ability of professionals to improve and adapt to advances in their profession. Students and residents may be able to accurately self-assess skills [11], but the ability to integrate the various components required to practice effective medicine is difficult to define and even more difficult to self-assess [12,13]. Physicians often fail to recognize what they do not know and the least experienced residents and physicians appear most likely to overrate their skills and knowledge [14-16]. Even experienced residents may not assess themselves as others would [17]. Self-assessment without comparison to some external standard such as an expert rater may not allow recognition of serious weaknesses, particularly in residents and physicians early in their careers [18]. However, the process of comparing self-assessments with external standards can only lead to improvement if the physician is made aware of discordance between his/her self-assessment and an assessment based on credible data and established standards [19,20].

The purpose of this study was to evaluate graduates' clinical competency, as perceived by the graduates themselves and by the experts. The outcome of this study will provide an evidence- based tool for identifying areas of strength and weakness in the curriculum as a basis for curriculum reforms and continuing professional development.

## Methods

### Study design

This is a cross-sectional observational study that constitutes a part of a larger study which deals with the different areas of medical graduates' competency: professionalism, communication skills, clinical skills, population health, management of information, and critical thinking. It was carried out during the period from 1^st^ January to 30^th^ March, 2010.

### Study setting

In Aden governorate secondary and tertiary care is provided both by public and private hospitals. Overall there are seven public hospitals with bed capacity ranging from 100 to 500 beds. For private hospitals, there are ten hospitals with bed capacity ranging from 50 to 100 beds. The study was carried out in the hospitals where the graduates were working at the time of data collection. For this purpose four public and four private hospitals were selected by convenient sampling method.

### Study population

#### Medical graduates

The study population covered all the medical graduates, Aden University (2005–2009), all of them have studied the same curriculum and were working at Aden hospitals, and gave their consent to participate in the study. A convenient sample of 105 graduates has covered all female and male graduates (61.9% females and 38.1% males) who were available at the time of data collection and fulfilled the inclusion criteria. No one of the graduates refused to participate in the study. The greater proportion of female participants among the study population does not reflect their actual size that does not exceed 45% of all the graduates during the study period, but is due to the fact that a large number of male graduates came from other regions of Yemen; and returned back to their regions after graduation.

#### Experts

The second group of the study population was expert evaluators who have direct observational knowledge of the graduates' competency in the operating environment; (being from the same department at the same hospital) and supervise their professional performance. The number of the experts was selected by a convenient sampling method according to the concentration of graduates in the hospital; each expert evaluated one or two medical graduates. For inclusion, the following criteria were adopted: a teaching staff in the faculty of medicine (assistant professor, associate professor or full professor) in clinical specialties; or a medical specialist with experience of not less than three years after getting the specialization certificate, and gave her/his informed consent to participate in the study. Those experts who fulfilled the above criteria were 63, distributed as follows: 24 females and 23 males from public hospitals and 3 females and 13 males from private hospitals.

### Tools for data collection

For both the graduates and experts, we used a self-administered questionnaire covering the different items of clinical competency. The questionnaire was elaborated following a thorough review of relevant literatures, particularly the global minimum essential requirements for medical education defined by the (IIME), (ABIM) and (ACGME). Content validity was examined by asking experts (10 experts) to judge whether the items cover all aspects of the domain intended to be measured in addition, a pilot study was done and the internal consistency reliability of the questionnaire was tested using (Cronbach's alpha) and was found to be >0.8. Both graduates and experts were asked to rate their perceived assessment on a five-point Likert scale ranging from very poor (being the lowest level of competency, scoring 1) to excellent (being the highest level, scoring 5). The final version of the questionnaire appears in Table 1.

**Table 1:** Questionnaire for the assessment of clinical competency by medical graduates and experts

**Clinical competency assessment**					
**Medical graduate is able to:**	**Excellent**	**V.G**	**Good**	**Poor**	**Very poor**
1. take an appropriate history including social issues such as occupational health					
2. perform a physical and mental status examination					
3. apply basic diagnostic and technical procedures, to analyze and interpret findings, and to define the nature of a problem					
4. perform appropriate diagnostic and therapeutic strategies with the focus on life-saving procedures and applying principles of best evidence medicine					
5. exercise clinical judgment to establish diagnoses and therapies					
6. recognize immediate life-threatening conditions.					
7. manage common medical emergencies					
8. manage patients in an effective, efficient and ethical manner including health promotion and disease prevention					
9. evaluate health problems and advise patients taking into account physical, psychological, social and cultural factors					
10. understand the appropriate utilization of human resources, diagnostic interventions, therapeutic modalities and health care facilities					

### Ethical considerations

1. The study protocol was approved by ''the Committee of Research and Postgraduate Studies, Faculty of Medicine and Health Science, Aden University'' which is responsible for both ethical and scientific review.

2. Permission was obtained from the authorities of the hospitals where the study was conducted.

3. Verbal consent was obtained from all potential participants after providing them with detailed explanation of the objectives, importance and benefits of the research. They were also assured that all the collected data would be handled with full confidentiality. Furthermore, they were informed that they had the right to refuse participation, and/or to withdraw at any moment.

### Data processing and analysis

Data processing was performed using the SPSS 16.0 software package. Multi items of clinical competency for each participant were computed into singular mean and singular percentage. For the convenience of analysis, the five-point Likert scale was re-categorized into three groups: 1- *inadequate* (which combined the poor and very poor scores), 2- *good* and 3- *very good* (which combined the very good and excellent scores).

The mean, frequency distribution, percentage of the variables were calculated; non-parametric Kruskal Wallis test for computed data was applied to verify whether the raters' assessments were influenced by variables such as age, gender, years of experience, work place, specialty and location of work at a (p ≤ 0.05) level of significance. A paired sample t- test was carried out to examine the difference between the means of the graduates and experts at a (p ≤ 0.05) level of significance.

## Results

### Graduates' self-assessment

The study covered 105 physicians (61.9% females and 38.1% males) who graduated during the period 2005–2009, and work in different public and private hospitals. The mean age was 28.8±2.30 years, ranging from 25 to 32 years. The experience of the graduates ranged from three months up to four years. The highest proportion of the participants in this study was those with four years experience 41.9% followed by those with experience of 2–3 years, 38.1%. The majority of the graduates 58.1% work in public hospitals, most of them work in the outpatient clinic 54.30%, and the remaining 45.7% work in the inpatient. The distribution of physicians in outpatient and in inpatient depends on the work load in different sites and is liable for change from time to time as perceived by the hospital administration. The majority of the graduates 47.6% work in internal medicine while the remaining work in pediatrics 21%, surgery 21% and gyn/obs 10.5% (Table 2).

**Table 2:** Socio-demographic characteristics of the graduates

**Characteristics**	**Male (n=40)**		**Female (n=65)**		**Total**	
	**No**	**%**	**No**	**%**	**No**	**%**
**Age group**						
25-27	11	34.4	21	65.6	32	30.5
28-30	13	31.0	29	69.0	42	40.0
>30	16	51.0	15	48.4	31	29.5
**Mean age**	29 years		28.6 years		28.8 years	
**SD**	±2.366		±2.114		±2.30	
**Experience (years)**						
<2	6	28.6	15	71.4	21	20.0
2-3	14	35.0	26	65.0	40	38.1
4	20	45.5	24	54.5	44	41.9
**Hospital**						
Public	20	32.8	41	67.2	61	58.1
Private	14	42.4	19	57.6	33	31.4
Both	6	54.4	5	45.5	11	10.5
**Specialty**						
Medicine	23	46.0	27	54.0	50	47.6
Surgery	14	63.6	8	36.4	22	21.0
Pediatrics	3	13.6	19	86.4	22	21.0
Gyn/Obs	0	.0	11	10.5	11	10.5
**location**						
Inpatient	16	40.0	32	49.2	48	45.7
Outpatient	24	60	33	50.8	57	54.3

SD: (standard deviation).

Table 3 exhibits graduates' self assessment of clinical competency according to gender, age-group, experience, hospital, specialty and location. It can be noticed that 12.4% of the graduates perceived their clinical competency as inadequate, while the remaining proportion were good and very good.

**Table 3:** Graduates' clinical competency self-assessment by gender, age-group, experience, hospital, specialty and location

**Variables**	**Assessment**								**df**	**(χ**^**2**^**)**	**p-value**
	**V.G**		**Good**		**Inadequate**		**Total**				
	**No**	**%**	**No**	**%**	**No**	**%**	**No**	**%**			
**Gender**											
Male	10	25.0	25	62.5	5	12.5	40	38.1	1	0.584	0.445
Female	22	33.8	35	53.8	8	12.3	65	61.9			
Total	32	30.5	60	57.1	13	12.4	105	100.0			
**Age group**											
25-27	8	25.0	22	68.8	2	6.2	32	30.5	2	0.015	.993
28-30	13	31.0	24	57.1	5	11.9	42	40.0			
>30	11	35.5	14	45.2	6	19.4	31	29.5			
**Experience (years)**											
<2	5	23.8	15	71.7	1	4.8	21	20.0	2	0.037	0.982
2-3	11	27.5	25	62.5	4	10.0	40	38.1			
4	16	36.4	20	45.5	8	18.2	44	41.9			
**Hospital**											
Public	16	26.2	36	59.0	9	14.8	61	58.1	2	3.005	0.224
Private	10	30.3	20	60.6	3	9.1	33	31.4			
Both	6	54.5	4	36.4	1	9.1	11	10.5			
**Specialty**											
Medicine	16	32.0	29	58.0	5	10.0	50	47.6	3	0.568	0.904
Surgery	6	27.3	12	54.5	4	18.2	22	21.0			
Pediatrics	6	27.3	14	63.6	2	9.1	22	21.0			
Gyn & Obs	4	36.4	5	45.5	2	18.2	11	10.5			
**Location**											
Inpatient	32	66.7	16	33.3	0	0.0	48	45.7	1	2.092	0.148
Outpatient	0	0.0	44	77.2	13	22.8	57	54.3			

df (degree of freedom).

Kruskal Walli test (H) =χ2.

With respect to gender, a higher proportion of the female graduates rated themselves as very good 33.8%, in comparison with only 25% of male graduates. On the other hand, the great majority of male graduates 62.5% believed that their clinical competency was good.

Though it was not statistically significant, there was a tremendous difference in the level of self-assessment between different age groups. More than 19% of the graduates in the age group > 30 years, perceived their clinical competency as inadequate, in contrast with only 6.2% of the graduates in the youngest age group i.e. 25–27 years. A similar result was noticed with respect to the relation of the graduates' years of experience with self-assessment. More than 18% of the graduates with 4 years of experience rated their clinical competency as inadequate, while only 4.8% of those with < 2 years of experience believed that their clinical competency was inadequate.

The hospital in which the graduates were working made a considerable difference in the competency rating level. The graduates who worked in private hospitals showed the highest percentage of good score 60.6%. On the other hand, graduates who worked in both public and private hospitals rated themselves as very good with 54.5%.

The main distinguishing feature in the location of work was that about two thirds of the graduates who worked in inpatient clinic 66.7% rated themselves as very good and 33.3% rated good. While those who worked in the outpatient clinic rated themselves as good 77.2% and inadequate 22.8%.

#### Expert assessment of medical graduates' competency

The study included 63 experts who have direct observational knowledge of the graduates' competency in the operating environment; (being from the same department at the same hospital) and supervise their professional performance.

Table 4 shows the socio-demographic characteristics of the experts. Among them were 57.1% males and 42.9% females. The mean age was 47±6.79 years, ranging from 33–64 years of age. The experts' experience ranged from 3 to 23 years. The highest proportion of the participants 58.7% belongs to those with 3–10 years experience. The majority of experts 46.0% are PhD holders, followed by the Master's degree and Board holders: 38.1% and 15.9% respectively.

**Table 4:** Socio-demographic characteristics of the experts

**Characteristics**	**Male (n=36)**		**Female (n=27)**		**Total (n=63)**	
	**No**	**%**	**No**	**%**	**No**	**%**
**Age group**						
33-45	13	50.0	13	50.0	26	41.3
46-55	15	51.7	14	48.3	29	46.0
>55	8	100.0	0	.0	8	12.7
**Mean age**	48.97		45		47	
**SD**	±7.15		±5.7		±6.797	
**Experience**						
3-10	18	48.6	19	51.4	37	58.7
11-15	7	53.8	6	46.2	13	20.6
>15	11	84.6	2	15.4	13	20.6
**Specialty**						
Medicine	11	64.7	6	35.3	17	27.0
Surgery	6	35.3	11	64.7	17	27.0
Pediatrics	17	85.0	3	15.0	20	31.7
Gyn+Obs	2	22.2	7	77.8	9	14.3
**Qualification**						
Msc	6	25.0	18	75.0	24	38.1
PhD	24	82.8	5	17.2	29	46.0
Board	6	60.0	4	40.0	10	15.9
**Position**						
Specialist	14	35.0	26	65.0	40	63.5
Assistant Professor	18	94.7	1	5.3	19	30.2
Associate professor	3	100.0	0	.0	3	4.8
Full Professor	1	100.0	0	.0	1	1.6
**Period of contact with graduate (years)**						
1	11	61.1	7	38.9	18	28.6
2	12	54.5	10	45.5	22	34.9
3	7	58.3	5	41.7	12	19.0
4	6	54.5	5	45.5	11	17.5

SD: (standard deviation).

The experts' assessment of graduate's clinical competency by gender, age-group, experience, hospital, specialty and location is shown in Table 5. As a whole, experts rated the graduates' competency as good 41% and very good 26.7%. Nevertheless, 32.4% of the graduates were assessed as inadequate.

**Table 5:** Expert assessment of medical graduate's clinical competency by gender, age-group, experience, hospital, specialty and location

**Variables**	**Assessment**								**df**	**χ**^**2**^	**p-value**
	**V.G**		**Good**		**Inadequate**		**Total**				
	**No**	**%**	**No**	**%**	**No**	**%**	**No**	**%**			
**Gender**											
Male	12	30.0	20	50.0	8	20.0	40	38.1	1	4.261	.04*
Female	16	24.6	23	35.4	26	40.0	65	61.9			
Total	28	26.7	43	41.0	34	32.4	105	100.0			
**Age group**											
25-27	7	21.9	12	37.5	13	40.6	32	30.5	2	7.413	.03*
28-30	10	23.8	14	33.3	18	42.9	42	40.0			
>30	11	35.5	17	54.8	3	9.7	31	29.5			
**Experience (years)**											
<2	4	19.0	7	33.3	10	47.6	21	20.0	2	3.121	.21
2-3	11	27.5	16	40.0	13	32.5	40	38.1			
4	13	29.5	20	45.5	11	25.0	44	41.9			
**Hospital**											
Public	14	23.0	24	39.3	23	37.7	61	58.1	2	7.614	.02*
Private	8	24.2	14	42.4	11	33.3	33	31.4			
Both	6	54.5	5	45.5	0	.0	11	10.5			
**Specialty**											
Medicine	12	24.0	23	46.0	15	30.0	50	47.6	3	1.825	.61
Surgery	5	22.7	10	45.5	7	31.8	22	21.0			
Pediatrics	4	18.2	10	45.5	8	36.4	22	21.0			
Gyn+Obs	7	63.6	0	.0	4	36.4	11	10.5			
**Location**											
Inpatient	10	20.8	18	37.5	20	41.7	48	45.7	1	3.459	.063
Outpatient	18	31.6	25	43.9	14	24.6	57	54.3			

df = (degree of freedom).

χ^2^: Kruskal Walli test.

*Significant p < 0.05.

Forty percent of female graduates were rated as inadequate in comparison with only 20% for male graduates. The difference in the rating level was statistically significant: *(p= 0.04)*. With regards to the age groups, more than 40% of the younger age groups (25–27 and 28–30 years) were rated as inadequate in comparison with only 9.7% in the age group >30 years. This difference was statistically significant: *(p= 0.03)*. With respect to the location of work, 41.7% of the graduates working in the inpatient were rated as inadequate in comparison with 24.6% of those working in outpatient.

The mean values of the graduates' clinical competency self-assessment compared with the experts' assessment are displayed in Table 6. Ten items covering the different dimensions of the graduates' clinical competency were assessed both by the graduates and the experts. As a whole, the graduates' self-assessment mean values were higher than those of the experts. The minimum mean values of assessment were 3.24 and 3.03 by the graduates and experts respectively, while the maximum mean values were 3.92 and 3.73.

**Table 6:** Graduates' clinical competency mean values self-assessment compared with experts' assessment

**Items**	**Assessment**		±**SD**	**(t)**	**p**
**Medical graduate is able to:**		**Mean**			
1. take an appropriate history including social issues such as occupational health	Graduates	3.70	.810	1.200	.233
	Experts	3.52	1.127		
2. perform a physical and mental status examination	Graduates	**3.64**	.856	2.375	.019*
	Experts	**3.30**	1.055		
3. apply basic diagnostic and technical procedures, to analyze and interpret findings, and to define the nature of a problem	Graduates	**3.55**	.855	2.194	.030*
	Experts	**3.28**	1.042		
4. perform appropriate diagnostic and therapeutic strategies with the focus on life-saving procedures and applying principles of best evidence medicine	Graduates	3.53	.833	1.453	.149
	Experts	3.33	1.174		
5. Able to exercise clinical judgment to establish diagnoses and therapies	Graduates	3.57	.745	.456	.650
	Experts	3.51	.991		
6. Able to recognize immediate life-threatening conditions.	Graduates	**3.80**	.801	2.646	.009*
	Experts	**3.44**	1.143		
7. manage common medical emergencies	Graduates	**3.92**	.851	2.608	.010*
	Experts	**3.61**	.814		
8. manage patients in an effective, efficient and ethical manner including health promotion and disease prevention	Graduates	3.79	. 829	.434	.665
	Experts	3.73	.963		
9. evaluate health problems and advise patients taking into account physical, psychological, social and cultural factors	Graduates	**3.69**	.870	2.136	.035*
	Experts	**3.38**	1.041		
10. understand the appropriate utilization of human resources, diagnostic interventions, therapeutic modalities and health care facilities	Graduates	3.24	.894	1.551	.124
	Experts	3.03	1.004		

SD: Standard deviation.

Paired sample T- Test.

* p: statistically significant <0.05.

We can observe that five out of ten items of the clinical competency showed high statistically significant difference between the graduates' self-assessment and the experts' assessment. Those items were:

1. Performing physical and mental status examination: *(p= 0.019)*.

2. Applying basic diagnostic and technical procedures to analyze and interpret findings, and to define the nature of a problem: *(p= 0.03)*.

3. Recognizing immediate life-threatening conditions: *(p= 0.009)*.

4. Managing common medical emergencies: *(p= 0.01)*.

5. Evaluating health problems and advising patients, taking into account physical, psychological, social and cultural factors: *(p= 0.035)*.

## Discussion

The assessment of graduates' performance is a very important tool to identify the strengths and weaknesses in medical education and to diagnose the curriculum situation.

Generally, this study revealed a wide discrepancy between the graduates' self- assessment and experts' assessment of the clinical competency of the graduates. While only 12.4% of the graduates perceived their clinical competency as inadequate, the experts rated more than 32% of the graduates as inadequate. This wide difference between experts' assessment and graduates' self-assessment might be explained by the tendency of the graduates to overestimates their abilities and competency. Similar findings were reported by Davis et al. and Su-Ting et al. [18,21].

With respect to self-assessment, the findings of this study revealed that more than 87% of the graduates rated themselves as very good and good; similar findings were reported by Moercke et al. [22] where 90% of the newly graduates assessed themselves as well prepared. In contrast, Ochsmann et al. [23] found that only 35% of junior doctors felt well prepared. This tremendous discrepancy might be explained by the different study design, different instrument for data collection and the different settings in which the studies were conducted.

On the other hand, 40% of female graduates were rated by experts as inadequate in comparison with only 20% of male graduates. This gross difference by gender might be explained by the socio-cultural context in which female doctors are frequently subject to an overload of responsibilities with family and child affairs, which might constitute a potential barrier standing in the way of adequate professional performance. Nevertheless, this issue requires further investigation. These findings were consistent with what has been reported by Kyoko Nomura et al. [24].

Though it was not statistically significant, there was a noticeable difference in the level of self-assessment between different age groups. More than 19% of the graduates in the age group > 30 years perceived their clinical competency as inadequate, in contrast with only 6.2% of the graduates in the youngest age group i.e. 25–27 years. A similar observation can be made with respect to the relationship between the graduates' years of experience and their self-assessment. More than 18% of the graduates with 4 years of experience rated their clinical competency as inadequate, in contrast with only 4.8% of those with < 2 years of experience. These findings are consistent with what has been reported by Ochsmann et al. and Morris et al. [23,25] and might be explained by the fact that as the graduates acquire more experience they become more critical and objective in the assessment of their professional performance.

On the other hand, great discrepancy was noted between self-assessment and experts' assessment with respect to age groups. While more than 40% of the younger age groups were rated by experts as inadequate, only 6.2% of the graduates rated themselves as inadequate. Similarly, we can notice that a large proportion 47.6% of the graduates with less years of experience was rated by experts as inadequate in contrast with only 4.8% for self-assessment. Similar findings were reported by Joshi et al. [26]. This finding supports the argument that graduates in general, and those of younger age groups and least experience, tend to overestimate their clinical skills and abilities.

The graduates feel that they are well prepared to deal with the main health problems presented in emergencies, as noted in the highest mean value 3.92 scored by the item "ability to *manage common medical emergencies*". This finding could be considered as a credit to the current curriculum which exposes the medical students to adequate opportunities for supervised practical training in this area during their clerkship and internship.

On the other hand, the graduates reported that they are not sufficiently confident in their competency in health management, as reflected in the lowest mean value 3.24 given to the item "the *ability to appropriate utilization of human resources, diagnostic interventions, therapeutic modalities and health care facilities"*. This finding corresponds with experts' assessment for this particular item of competency; hence, it constitutes an alarming signal for curriculum planners and requires further investigations. Similar findings were reported by Silber et al. [27].

A remarkable observation in the findings of this study was that both graduates and experts gave relatively high mean values (3.79 and 3.73 respectively) for the competency to *"manage the patients in an effective, efficient and ethical manner including health promotion and disease prevention*". This item of clinical skills acquires great importance in medical education, as it embraces the essence of professionalism in the clinical practice and patient management, and gives credit to the current curriculum.

## Conclusion

There was a wide discrepancy between the graduates' self-assessment and experts' assessment, particularly in the level of inadequate performance. Graduates in general, and those of younger age groups and least experience in particular, tend to overestimate their clinical skills and competency. Both graduates and experts were in agreement that the graduates have adequate competency to manage the patients ethically in an effective and efficient manner. Both graduates and experts agreed that competency in the management of human resources and health care facilities were at a marginal level and needs further improvement. Feedback with the findings of this study for the graduates, experts (evaluators) and curriculum planners is essential for curriculum reforms that should address the identified areas of competency that need further improvement as well as for continuing professional development programs.

Self-assessment should be conducted on regular basis, at least once per year with feed back for all stakeholders in order to make the necessary interventions for promoting the professional competency and the quality of care. Further studies on larger samples and different settings are recommended.

## Competing interest

The authors declare that they have no competing interests.

## Authors' contributions

FTA was involved in the conception, design, analysis and interpretation of data, report writing and manuscript writing. ASH was involved in the conception, design and manuscript review. Both authors have read and approved the final version of the manuscript.

## Pre-publication history

The pre-publication history for this paper can be accessed here:

http://www.biomedcentral.com/1472-6920/13/24/prepub
